# Multimodality Treatment Including Triplet Regimen as First-Line Chemotherapy May Improve Prognosis of Serum AFP-Elevated Gastric Cancer with Liver Metastasis

**DOI:** 10.1155/2017/5080361

**Published:** 2017-12-24

**Authors:** Yakun Wang, Lin Shen, Ming Lu, Zhi Ji, Xiaotian Zhang

**Affiliations:** Department of Gastrointestinal Oncology, Key Laboratory of Carcinogenesis and Translational Research (Ministry of Education), Peking University Cancer Hospital & Institute, No. 52, Fucheng Road, Haidian District, Beijing 100142, China

## Abstract

Serum *α*-fetoprotein- (AFP-) elevated gastric cancer is a rare tumor that has a poor prognosis due to high incidence of liver metastasis. This study sought to investigate the optimal treatment modality. A total of 319 gastric cancer patients with liver metastasis (GCLM) whose serum AFP levels were tested before treatment were enrolled in this study. They were classified as the serum AFP ≥ 20 ng/ml group (*n* = 74) and the AFP < 20 ng/ml group (*n* = 245). Median OS of the AFP < 20 ng/ml group was significantly longer than that of the AFP ≥ 20 ng/ml group (15.7 m versus 10.9 m, *P* = 0.004). ORR of first-line chemotherapy was 43.3% and 56.1% of the two groups, respectively (*P* = 0.024). Of patients who received doublet regimen, ORR of the AFP ≥ 20 ng/ml group was significantly lower (38.2 versus 56.9%, *P* = 0.013), while in those received triplet regimens, ORR between two groups was similar (66.7% versus 66.7%, *P* = 0.676). Moreover, for patients of the AFP ≥ 20 ng/ml group, those who reached PR had a longer survival period (15.4 m versus 9.4 m, *P* = 0.017), and combined with local treatment for liver metastasis also seemed to improve prognosis (19.2 m versus 8.4 m, *P* = 0.003). In conclusion, serum AFP-elevated GCLM had a poorer prognosis. Multimodality treatment including aggressive first-line chemotherapy with triplet regimen may be needed when treating them.

## 1. Introduction

Serum *α*-fetoprotein (AFP) has been proposed as a tumor marker for screening liver tumor and germ cell tumors in the clinic [1–3]. 70–95% of hepatocellular cancers are associated with increased AFP level. Serum AFP-elevated gastric cancer was first reported by Alpert et al. in 1970 [4]. Many other reports followed. The incidence of AFP-producing gastric cancer was merely 1.3–15.0% worldwide [5]. In most literatures, the gastric cancer patients with serum AFP elevation were found to have doughty invasiveness and poor prognosis [6–9]. Also, Liu et al. reported that the dismal prognosis of serum AFP-elevated gastric cancer was mainly due to high incidence of synchronous and metachronous liver metastasis, even when radical operation was done [5]. Therefore, systemic chemotherapy became the predominant treatment method for serum AFP-elevated gastric cancer with liver metastasis (GCLM).

Due to the rarity of this special cancer, there is limited data in the literature about optimal treatment modality. Although previous studies reported that conventional chemotherapy was predominantly ineffective for these patients [10, 11], it remains controversial whether to perform systemic chemotherapy for this subtype of GCLM, and there had been so far no suggestion for choosing the optimal regimen.

The potential underlying molecular mechanism of AFP-producing gastric cancer may be the common embryonic origin of the stomach and liver from the foregut [12]. Koide et al. found that AFP-related gastric cancers had higher proliferative activity, weaker apoptosis, and richer neovascularization, compared with that of AFP-negative gastric cancers [13]. As the precise underlying mechanism of serum AFP-elevated GC remains to be elucidated, the optimal treatment approach requires further consideration. We aim to discover the optimal treatment modality for this special subtype GCLM.

## 2. Patients and Methods

### 2.1. Patient Selection

Between 2005 and 2016, 2047 patients were diagnosed with advanced gastric adenocarcinoma in our institute. 516 of them were diagnosed with liver metastasis (LM), including postoperative LM and LM at the initial diagnosis. We included subjects who had serum AFP test result before treatment, leaving 319 patients for analysis. Pretreatment serum AFP was assessed by radioimmunoassay (normal value: <7.0 ng/ml).

### 2.2. Data Collection

We collected age, gender, ECOG, primary lesion site, histological type, Lauren classification, human epidermal growth factor receptor-2 (HER2) status, serum AFP level before treatment, first-line chemotherapy regimens, response, local treatment for LM, and survival information.

### 2.3. Follow-Up Care

All patients were regularly followed up from the date of the first hospitalization at our center. Objective response rate (ORR) were evaluated by RECIST version 1.0 (before 2009) and RECIST version 1.1, and severe adverse events (≥grade 3) were recorded. Overall survival (OS) was defined as the time from inspection of liver metastasis to death from any cause or last follow-up.

### 2.4. Statistics

The Pearson chi-square test was used to measure the differences between variables. The Fisher exact test was used when the numbers were less than five. To identify prognostic factors of overall GCLM patients and the AFP ≥ 20 ng/ml subgroup, survival durations were calculated using the Kaplan-Meier method and Cox regression. For all tests, a *P* value < 0.05 was defined as significant. The SPSS software program (version 21.0; SPSS, Chicago, Illinois) was used for the analyses. The GraphPad Prism 6 (GraphPad Software Inc., La Jolla, CA) was used for chart making.

## 3. Results

### 3.1. Characteristics of GCLM of the AFP ≥ 20 ng/ml Group and AFP < 20 ng/ml Group

Of the 319 eligible patients, 74 (23.2%) were found to have serum AFP ≥ 20 ng/ml.  Table 1 compared the clinicopathologic features of patients between the AFP ≥ 20 ng/ml group (*n* = 74) and AFP < 20 ng/ml group (*n* = 245). Results of age, gender, ECOG, disease status, primary lesion site, Lauren classification, HER2 status, peritoneal metastasis, and number of LM were similar between two groups.

Notably, compared with the serum AFP < 20 ng/ml group, 10 (13.5%) patients were diagnosed with hepatoid adenocarcinoma in the AFP ≥ 20 ng/ml group. Gastric hepatoid adenocarcinoma (GHA) was defined as a special subtype of primary gastric adenocarcinoma characterized by the histologic structures of “hepatocellular carcinoma- (HCC-) like differentiation” with or without excessive production of AFP [14, 15].

In addition, portal vein tumor thrombus (PVTT) occurred frequently in the AFP ≥ 20 ng/ml group, while it is rarely observed in the AFP < 20 ng/ml group (14.9% versus 2.0%, *P* < 0.001). The clinicopathologic features of the two groups were detailed in Table 1.

### 3.2. Treatment Modality and Response to First-Line Chemotherapy between GCLM of the AFP ≥ 20 ng/ml Group and AFP < 20 ng/ml Group

In the analysis of first-line chemotherapy regimens, for the AFP ≥ 20 ng/ml group, 46 (62.2%) received platinum-based doublet regimen, including oxaliplatin + capecitabine in 23 patients, oxaliplatin + S-1 in 8 patients, cisplatin + capecitabine in 9 patients, cisplatin + S-1 in 2 patients, oxaliplatin + 5-FU in 3 patients, and cisplatin + 5-FU in 1 patient. 13 (17.6%) received taxane-based doublet regimen, including paclitaxel + capecitabine in 10 patients, paclitaxel + S-1 in 1 patient, paclitaxel + 5-FU in 1 patient, and docetaxel + capecitabine in 1 patient. 9 (12.2%) received triplet regimen (specific regimens were shown in Table 5), and 6 (8.1%) received single-drug regimen (including paclitaxel, S-1, and capecitabine).

For the AFP < 20 ng/ml group, 156 (63.7%) received platinum-based doublet regimen, including oxaliplatin + capecitabine in 68 patients, oxaliplatin + S-1 in 30 patients, cisplatin + capecitabine in 41 patients, cisplatin + S-1 in 5 patients, oxaliplatin + 5-FU in 8 patients, and cisplatin + 5-FU in 4 patients. 44 (18.0%) received taxane-based doublet regimen, including paclitaxel + capecitabine in 28 patients, paclitaxel + S-1 in 10 patients, paclitaxel + oxaliplatin in 2 patients, docetaxel + 5-FU in 1 patient, and docetaxel + oxaplitatin in 3 patients. Also, there were 17 (6.9%) patients who received triplet regimen (combination of platinum, taxanes, and fluorouracil drugs), and 19 (7.8%) received single-drug regimen (including paclitaxel, S-1, and capecitabine). Analysis showed no significant differences between two groups.

Among the original 319 patients, there were 68 (93.2%) and 189 (77.1%) patients evaluable for their response to first-line chemotherapy in the two groups, respectively. Compared with the AFP < 20 ng/ml group, overall objective response rate (ORR) to first-line chemotherapy was significantly lower in the AFP ≥ 20 ng/ml group (41.2% versus 56.1%, *P* = 0.024).

With regard to second-line chemotherapy, there were fewer patients of the AFP ≥ 20 ng/ml group who received second-line chemotherapy than the AFP < 20 ng/ml group (40.0% versus 53.0%, *P* = 0.055). Regimens mainly involved taxanes and fluorouracil. Moreover, there were 1 (1.4%) and 8 (3.3%) patients who received surgery treatment after first-line chemotherapy in the two groups. In addition, 23 (31.1%) and 60 (24.5%) patients received local treatment for LM in the two groups, respectively, and there were no significant differences between them, either. Comparison of treatments and response between two groups were shown in Table 2.

### 3.3. Objective Response Rate (ORR) of Doublet and Triplet Regimens between the AFP ≥ 20 ng/ml Group and AFP < 20 ng/ml Group

Table 3 summarized the response to doublet and triplet regimen between two groups. Result showed that compared with the AFP < 20 ng/ml group, the AFP ≥ 20 ng/ml group had a significantly poor response to platinum/taxane-based doublet regimen (38.2% versus 56.9%, *P* = 0.013). However, with chemotherapy of triplet regimen, ORR was similar between two groups (66.7% versus 66.7%, *P* = 0.676).

We further compared ORR and occurrence of severe adverse events of different regimens in the AFP ≥ 20 ng/ml group in Table 4. Result showed that ORR of triplet regimen was higher than doublet regimen (66.7% versus 25.0–43.9%), but analysis showed no significance (*P* = 0.162). Notably, ORR of taxane-based doublet regimen was especially low (25.0%) .

In the analysis of adverse events, triplet regimen showed a significantly higher rate of ≥grade3 adverse events (66.7% versus 22.0–25.0%, *P* = 0.014).

### 3.4. Case by Case Analysis of Nine GCLM Patients with AFP ≥ 20 ng/ml Who Received Triplet Regimen as First-Line Chemotherapy

In the AFP ≥ 20 ng/ml group, nine patients received triplet regimen as first-line chemotherapy. Among them, six were male, and only one patient was more than 60 years old. Serum AFP levels ranged from 22 ng/ml to 208,072 ng/ml. One patient's primary tumor was located at GEJ, others' primary tumor was located at gastric body or antrum. Three patients were diagnosed with hepatoid adenocarcinoma, the six left were common adenocarcinoma. Of five cases that Lauren's classification and HER2 status were known, one case was with intestinal type, two with diffuse type, and two with mixed type, only one case was examined as HER2 positive.

Two-thirds of patients (6/9) achieved PR after triplet regimen as first-line chemotherapy, and two achieved SD with tumor shrinkage of 18%. Only one patient's disease progressed quickly after only one cycle of chemotherapy. Two-thirds of patients (6/9) suffered from ≥grade 3 adverse events, and four of them had to change treatment regimens because of intolerable toxicity. Disease-free survival (DFS) and overall survival (OS) data were also included in Table 5.

### 3.5. Prognostic Factors of Overall GCLM and GCLM with Serum AFP ≥ 20 ng/ml

**Figure 1:**
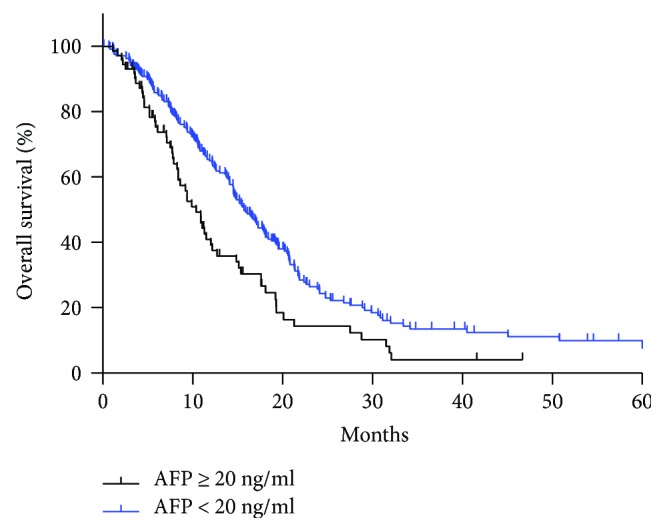
The median OS of group 1 (AFP ≥ 20 ng/ml GCLM) and group 2 (AFP < 20 ng/ml GCLM) was 10.9 m and 15.7 m, respectively (*P* = 0.002).

Median serum AFP level was 480.9 ng/ml and 3.1 ng/ml in the AFP ≥ 20 ng/ml group and AFP < 20 ng/ml group, respectively. Median overall survival period was 10.9 m and 15.7 m in the two groups (*P* = 0.004, Figure 1). Furthermore, multivariate analysis revealed that besides typical prognostic factors of histologic type, extrahepatic unresectable advanced/metastatic sites, response to chemotherapy, and so on, elevation of serum AFP was also an independent prognostic factor for overall GCLM (details were shown in Table 6).

**Figure 2:**
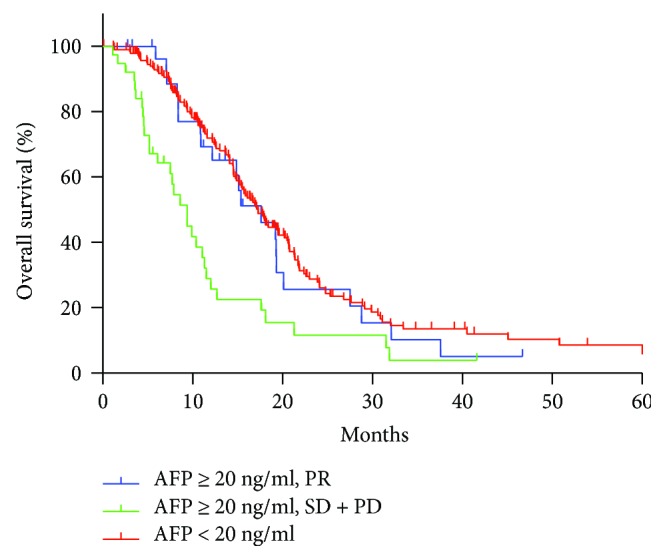
The median OS of group 1 (AFP ≥ 20 ng/ml and reached PR after first-line chemotherapy), group 2 (AFP ≥ 20 ng/ml and achieved SD/PD after first-line chemotherapy), group 3 (AFP < 20 ng/ml) was 17.6 m, 9.4 m, 17.3 m, respectively (*P* < 0.001).

**Figure 3:**
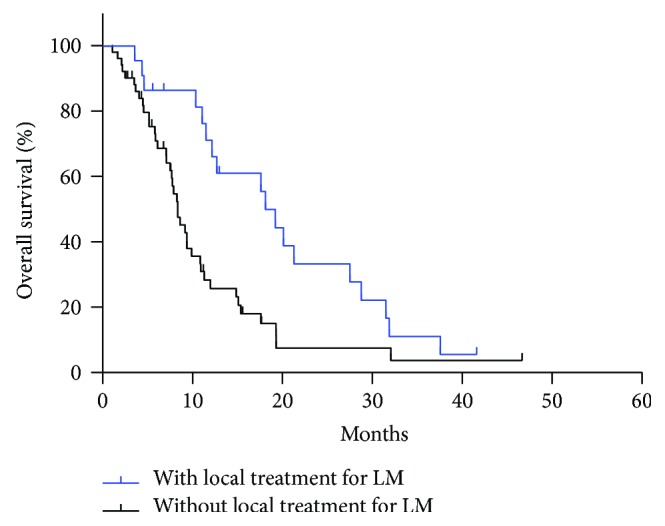
In serum AFP ≥ 20 ng/ml GCLM, the median OS of group 1 (with local treatment for LM) and group 2 (without local treatment for LM) was 19.2 m and 18.3 m, respectively (*P* = 0.003).

We further investigated the prognostic factors of GCLM patients with serum AFP ≥ 20 ng/ml. While the ORR of triplet regimen was excellent in AFP-elevated GCLM, analysis showed no significant difference in survival between doublet and triplet regimens (37.6 m versus 9.9 m, *P* = 0.157) due to the rather small number (9/74, 12.2%) of patients receiving triplet regimens. In addition, for GCLM patients with serum AFP ≥ 20 ng/ml, univariate and multivariate analysis revealed that response to first-line chemotherapy was an independent prognostic factor. Patients reached PR had a better prognosis, similar to overall population, while patients evaluated as SD/PD had the worst survival prognosis (*P* < 0.001, Figure 2). Also, survival analysis showed that combined with local treatment for LM may result in better prognosis and significant difference exist (19.2 m versus 8.4 m, *P* = 0.003) (Figure 3).

## 4. Discussion

The main findings of this study are as follows: (1) serum AFP ≥ 20 ng/ml GCLM showed a poorer prognosis than the AFP < 20 ng/ml group. (2) Doublet regimen was significantly less effective for the AFP ≥ 20 ng/ml group than in the AFP < 20 ng/ml group. (3) Triplet regimen increased ORR compared to doublet regimen when treating serum AFP ≥ 20 ng/ml GCLM, but result showed no significance on survival.

AFP is a fetal serum protein by fetal and yolk sac cells and by some fetal gastrointestinal cells [16]. After birth, the level of AFP rapidly decreased. The elevation of AFP in serum of people older than one year is indicative of either HCC or yolk sac tumor. In addition, some reports showed that AFP could also be produced by other tumors, including gastric cancer, rectal cancer, pancreas cancer, gallbladder cancer, lung cancer, and bladder cancer [17].

In 1970, Alpert et al. first reported a case of AFP-producing GC, which refers to a type of gastric cancer that AFP is positive in the immunohistochemical staining of pathological specimen [4]. In 1985, Ishikura et al. proposed a new entity, hepatoid adenocarcinoma of the stomach, which showed a histologic appearance typical of HCC, including solid, trabecular, and pseudogranular structure, tumor cells were round or polygonal in shape [18]. In addition, Nagai et al. clarified that hepatoid adenocarcinoma of the stomach had characteristic histologic features and a poor prognosis irrespective of AFP production and should be distinguished from AFP-positive GC without hepatoid features [14]. However, due to focal distribution and high heterogeneity of gastric hapatoid adenocarcinoma (GHA) [9, 19], almost all GHA cases reported in previous literatures were diagnosed from postoperative specimens. On the other hand, due to aggressive behavior and high frequency of liver metastasis [8], most patients had lost operation opportunity at diagnosis. Thus, most of our patients' feature was just with serum AFP elevation, with only ten patients diagnosed as GHA. The definition of AFP-producing GC varies between studies owing to difficulty in setting the cut-off value; considering liver metastasis can be a factor for mild increase in AFP level, we chose AFP ≥ 20 ng/ml as a cut-off value in this study.

Serum AFP-elevated gastric cancer is rare, only accounts for 2.3–7.1% of all gastric cancers [6, 20], but in GCLM population, our result showed that 23.2% (73/319) patients' serum AFP exceeded 20 ng/ml. To clarify the variables associated with the poor prognosis of the AFP ≥ 20 ng/ml group, we next reviewed the data of patients and analyzed the differences between two groups. Result showed the serum AFP ≥ 20 ng/ml group had a significantly poorer response to first-line chemotherapy in comparison to the AFP < 20 ng/ml group. Also, survival analysis revealed that response to first-line chemotherapy was significantly associated with survival prognosis, and for the AFP ≥ 20 ng/ml group, those who reached PR after first-line chemotherapy had a similar survival period as those AFP < 20 ng/ml. These results indicate that choosing effective chemotherapy regimen may improve prognosis of serum AFP-elevated GCLM.

In general treatment of inoperable locally advanced and/or metastatic (stage IV) GC, doublet combinations of platinum and fluropyrimidines are generally used, with an overall ORR of 52.2–58.7% [21, 22]. There remains controversy regarding the utility of triplet regimes, especially in China and Japan [23]. Although there is considerable improvement in medicine science, serum AFP-elevated GC is found to have a poor response to chemotherapy and thus associated with a poor prognosis [24]; basic research indicated that AFP-producing cell lines were not sensitive to many drugs [25]. This clinical study further suggest that this special subtype of gastric cancer may be less sensitive to doublet regimen including platinum and fluoropyrimidines, which was in accordance with a previous study reported that for GHA, ORR and disease control rate (DCR) to palliative chemotherapy was only 7.7% (1/13) and 45.1% (6/13), respectively [24]. However, by comparing ORR and AEs of different regimens used in the AFP ≥ 20 ng/ml group, we found that triplet regimens combining platinum, taxanes, and fluoropyrimidines achieved a satisfactory ORR in this special subtype of GCLM. Although ≥grade 3 adverse events were reported in 66.7% patients, they were all reversible and there was no treatment-related death occurred.

Despite the excellent ORR of triplet regimen, median OS was similar in patients who received triplet regimen and doublet regimen. This phenomenon that could mainly attribute to the rate of patients who received triplet regimen was relatively low in our study (9/73), and four of nine patients were still alive until the last follow-up. On the other hand, in clinic practice, triplet regimen was always used in patients with heavy tumor burden, which may be associated with poor prognosis as well. Case by case analysis revealed there were two patients who lived longer than two years. Both of them reached PR after triplet regimen chemotherapy. One of them was examined as HER2 positive and received Herceptin treatment at second-line chemotherapy. In addition, the patient also received TACE for LM, which may also improve survival prognosis of gastric cancer with liver metastasis [11, 26]. The other case was diagnosed as GHA, who also received multiline systemic treatment, including apatinib. We noted that there were two patients complicated with PVTT, which is a special characteristic of AFP-related gastric cancer [27]; a high rate of PVTT in AFP-producing gastric cancer (14.9% in our study) may indicate high intendancy of vascular invasion and angiogenesis [28, 29]. It was reported that silencing AFP inhibits VEGF production in human HCC cells [30]. The function of apatinib, as a small molecular tyrosine kinase targeting VEGF-R2 (vascular endothelial growth factor receptor-2), is antiangiogenesis. There is another case report of targeted therapy with apatinib in a patient with advanced gastric cancer and high serum level of AFP and PFS achieved five months [31]. Thus, the inhibitors of VEGF or VEGFR might become potential drugs to treat this special subtype of gastric cancer. This long-time survivor in our study also received TACE for LM during treatment process.

We also showed that the only patient resistant to triplet regimen was a young female, with diffuse type Lauren classification and peritoneal metastasis and ascites, which were associated with poor prognosis and bad response to conventional systemic chemotherapy [32]. Furthermore, in our study, almost half of patients (cases 1, 4, 7, and 8) had to stop triplet regimen during treatment process because of intolerable adverse events; thus, severe toxicity of triplet regimen may also attribute to poor survival prognosis, and optimizing triplet regimens deserves further study.

In a word, survival analysis and the two successfully treated cases indicate that although AFP-producing gastric cancer is often advanced and complicated with liver metastasis, long-term survival can be achieved by multimodality treatment including triplet regimen chemotherapy; those had a PR response of first-line chemotherapy could get more chance to be treated.

## 5. Conclusions

Serum AFP-elevated gastric cancer is a small subgroup of gastric carcinoma with high metastatic potential to the liver and poor prognosis. Multimodality treatment including aggressive chemotherapy of triplet regimen may be worthwhile to improve prognosis of serum AFP-elevated GCLM; better tolerated regimens should be investigated further in the future.

## 6. Shortcomings and Perspectives

Although the retrospective nature of this study and the number of cases treated with triplet regimen were relatively small, the results could still provide some clinical value. With such rare tumors, for which large clinical trials are not feasible, it became very important to summarize clinical experience retrospectively. Not limited within GCLM, maybe triplet regimen can be tried to be used in all AFP-elevated gastric cancer in future clinical practice. Although this type of gastric cancer is rare, it deserves further studies.

## Figures and Tables

**Table 1:** Comparison of characteristics of GCLM between the AFP ≥ 20 ng/ml group and AFP < 20 ng/ml group.

Variable	AFP ≥ 20 ng/ml (*n* = 74)	AFP < 20 ng/ml (*n* = 245)	*P*
Gender			
Male	57 (77.0%)	198 (80.8%)	0.288
Female	17 (23.0%)	47 (19.2%)	
Age (years)			
≥65	56 (75.7%)	165 (67.3%)	0.111
<65	18 (24.3%)	80 (32.7%)	
ECOG			
0-1	66 (89.2%)	220 (89.8%)	0.514
2-3	8 (10.8%)	25 (10.2%)	
Disease status			
LM after radical resection	10 (13.5%)	45 (18.4%)	0.216
LM at first diagnosis	64 (86.5%)	200 (81.6%)	
Primary lesion site			
GEJ	24 (33.3%)	101 (42.4%)	0.385
Non-GEJ	48 (66.7%)	137 (57.6%)	
Unknown	2	7	
Histological type			
Well differentiated^a^	23 (31.5%)	94 (39.3%)	<0.001
Poorly differentiated^b^	40 (54.8%)	145 (60.7%)	
GHA	10 (13.7%)	0 (0%)	
Unknown	1	6	
Lauren classification			
Intestinal type	34 (70.8%)	103 (65.6%)	0.633
Diffused type	5 (10.4%)	25 (15.9%)	
Mixed type	9 (18.8%)	29 (18.5%)	
Unknown	26	88	
HER2 status			
Positive	13 (24.5%)	54 (30.9%)	0.240
Negative	40 (75.5%)	121 (69.1%)	
Unknown	21	70	
Peritoneal metastasis			
Yes	9 (12.2%)	38 (15.5%)	0.321
No	65 (87.8%)	207 (84.5%)	
Number of LM			
1–3	12 (16.2%)	48 (20.3%)	0.367
>3	62 (83.8%)	188 (80.7%)	
PVTT			
Yes	11 (14.9%)	5 (2.0%)	<0.001
No	63 (85.1%)	240 (98.0%)	

^a^Including well-differentiated and moderately differentiated adenocarcinoma. ^b^Including poorly differentiated and signet ring cell adenocarcinoma. GHA = gastric hepatoid adenocarcinoma; ECOG = Eastern Cooperative Oncology Group; GEJ = gastroesophageal junction; HER2 = human epidermal growth factor receptor-2; AFP = *α*-fetoprotein; LM = liver metastasis; PVTT = portal vein tumor thrombus.

**Table 2:** Comparison of treatments and response in GCLM between the AFP ≥ 20 ng/ml group and AFP < 20 ng/ml group.

Variables	AFP ≥ 20 ng/ml	AFP < 20 ng/ml	*P*
First-line chemotherapy regimens			
Platinum-based doublet regimen	46 (62.2%)	156 (63.7%)	0.325
Taxane-based doublet regimen	13 (17.6%)	44 (18.0%)	
Triplet regimen	9 (12.2%)	17 (6.9%)	
Single-drug regimen	6 (8.1%)	19 (7.8%)	
Others	0 (0.0%)	9 (3.7%)	
Response of first-line chemotherapy			
PR	28 (41.2%)	106 (56.1%)	0.024
SD + PD	40 (58.8%)	83 (43.9%)	
Subsequent therapies after the first-line chemo			
Second-line chemotherapy			
Yes	24 (40.0%)	97 (53.0%)	0.055
No	36 (60.0%)	86 (47.0%)	
Surgery treatment			
Yes	1 (1.4%)	8 (3.3%)	0.344
No	73 (98.6%)	237 (96.7%)	
Local treatment of LM^a^			
Yes	23 (31.1%)	60 (24.5%)	0.163
No	51 (68.9%)	185 (75.5%)	

^a^Including TACE, ablation, radiotherapy, and liver resection. PR = partial response; SD = stable disease; PD = progressive disease; TACE = transcatheter arterial chemoembolization.

**Table 3:** Comparison of ORR of different chemotherapy regimens between two groups.

Regimens	AFP ≥ 20 ng/ml	AFP < 20 ng/ml	*P*
Platinum/taxane-based doublet regimen			
PR	21 (38.2%)	91 (56.9%)	0.013
SD + PD	34 (61.8%)	69 (43.1%)	
Triplet regimen			
PR	6 (66.7%)	8 (66.7%)	0.676
SD + PD	3 (33.3%)	4 (33.3%)	

**Table 4:** ORR and severe AEs of different regimens in the AFP ≥ 20 ng/ml group.

ORR and AEs	Platinum-based doublet regimen (*n* = 41)	Taxane-based doublet regimen (*n* = 12)	Triplet regimen (*n* = 9)	*P*
PR	18 (43.9%)	3 (25.0%)	6 (66.7%)	0.162
SD + PD	23 (56.1%)	9 (75.0%)	3 (33.3%)	
≥grade 3 AEs	9 (22.0%)	3 (25.0%)	6 (66.7%)	0.014

AE = adverse event; OS = overall survival.

**(a):** 

Age/sex	Primary lesion site	Histological type	Serum AFP level (ng/ml)	Lauren classification	HER2 status	PVTT	Peritoneal metastasis
34/M	Body	GHA	455	Intestinal	Negative	No	No
59/M	Antrum	GHA	208,072	NK	NK	No	No
43/M	Body	Poorly differentiated adenocarcinoma	7307	NK	NK	Yes	No
58/M	Antrum	Poorly differentiated adenocarcinoma	113	Mixed	Negative	No	No
39/F	Body	Poorly differentiated adenocarcinoma	3042	Diffuse	Negative	No	Yes
56/F	Antrum	Middle-differentiated adenocarcinoma	22	Intestinal	Positive	No	No
75/F	Antrum	Middle-differentiated adenocarcinoma	131	NK	NK	No	No
59/M	Body	Middle-differentiated adenocarcinoma	2108	NK	NK	No	No
42/M	GEJ	GHA	868	Mixed	Negative	Yes	No

**(b):** 

Regimen	Cycles	Evaluation	PFS (m)	≥grade 3 toxicity	Other treatments	OS (m)	Follow-up status
PCF	4	PR	9.5	BWL	FOLFIRINOX; olaparib; apatinib; PD-1 antibody; TACE	22.8	Alive
PCF	6	PR	4.2	Vomiting	No	7.1	Dead
POS	5	SD	6.0	No	No	11.0	Alive
POS	5	PR	6.2	Hematological; sensory neuropathy	Radiotherapy	8.0	Alive
DCF	1	PD	0.8	No	No	1.7	Dead
ECF	6	PR	6.1	No	Herceptin; TACE	37.6	Dead
DCF	4	PR	3.4	Hematological; vomiting; mucosal reaction	No	10.9	Dead
PCF	3	PR	2.6	Cardiac toxicity	Gastrectomy	10.9	Dead
POS	5	SD	5.83	Hematological	Gastrectomy; liver resection; apatinib; PD-1 antibody	13.6	Alive

F = female; M = male; NK = not known; BWL = body weight loss; PVTT = portal vein tumor thrombus; PCF = paclitaxel + cisplatin+ 5-fluorouracil; POS = paclitaxel + oxaplatin + S-1; DCF = docetaxel + cisplatin+ 5-fluorouracil; ECF = epirubicin + cisplatin+ 5-fluorouracil.

**Table 6:** Univariate and multivariate analysis of survival outcomes in overall GCLM and of the subgroup of serum AFP ≥ 20 ng/ml.

	GCLM with serum AFP ≥ 20 ng/ml (*n* = 74)					Overall GCLM (*n* = 319)				
	Univariate analysis (KM)		Multivariate analysis (Cox)			Univariate analysis (KM)		Multivariate analysis (Cox)		
Variable	mOS (m)	*P* value	HR	95% CI	*P* value	mOS (m)	*P* value	HR	95%CI	*P* value
Gender										
Male	9.9	0.608				14.1	0.609			
Female	15.1					14.8				
Age										
≤65	9.2	0.773				14.8	0.379			
>65	11.3					14.9				
ECOG										
0-1	9.2	0.095				16.5	0.227			
2-3	11.3					14.5				
Disease status										
LM after radical resection	10.4	0.411				19.3	0.046	0.700	0.359–1.364	0.295
LM at first diagnosis	10.9					14.5				
Primary lesion site										
GEJ	14.9	0.103				15.2	0.600			
Non-GEJ	9.4					14.5				
Histologic classification										
Intestinal	12.7	0.757				16.1	0.021	0.558	0.370–0.840	**0.005**
Nonintestinal	12.0					11.2				
HER2 status										
Positive	12.7	0.888				17.3	0.293			
Negative	15.1					15.2				
Extrahepatic M										
Present	10.4	0.116				14.6	0.952			
Absent	12.2					15.5				
Peritoneal M										
Present	4.6	<0.001	4.411	1.817–10.712	**0.001**	10.8	0.001	1.953	1.105–3.451	**0.021**
Absent	11.3					15.4				
LM numbers										
1–3	17.6	0.115				19.5	0.002	0.416	0.236–0.733	**0.002**
>3	9.9					12.7				
First-line chemo regimen										
Doublet regimen	9.9	0.157				14.9	0.816			
Triplet regimen	37.6					17.3				
Response to first-line chemo										
PR	15.4	0.017	0.328	0.173–0.624	**0.001**	19.2	<0.001	0.506	0.334–0.768	**0.001**
SD + PD	9.4					11.4				
Local treatment for LM										
Yes	19.2	0.003	0.356	0.179–0.710	**0.003**	20.8	<0.001	0.623	0.394–0.986	**0.043**
No	8.4					12.4				
Serum AFP level (ng/ml)										
≥20 ng/ml						10.9	0.004	1.553	1.006–2.397	**0.047**
<20 ng/ml						15.7				
